# Social Health Programming During Adolescence Is Associated with Increased Serum Levels of Carotenoids, Vitamin A, and Vitamin E in Young Women: An Observational Cohort Study

**DOI:** 10.3390/antiox15040498

**Published:** 2026-04-16

**Authors:** Rebecca Drakowski, Matthew VanOrmer, Laura Ebers, Katie Mayhan, Anum Akbar, Colman Freel, Taija Hahka, Rebekah A. S. Rapoza, Corrine Hanson, Keyonna M. King, Aaryn Mustoe, Melissa K. Thoene, Ann L. Anderson-Berry

**Affiliations:** 1 Department of Pediatrics, University of Nebraska Medical Center, Omaha, NE 68198, USAtaija.hahkavermeulen@bhsu.edu (T.H.); melissak.thoene@unmc.edu (M.K.T.);; 2 Medical Nutrition Education Program, College of Allied Health Professions, University of Nebraska Medical Center, Omaha, NE 68198, USA; 3 Department of Health Promotion, University of Nebraska Medical Center, Omaha, NE 68198, USA; 4 Southwest National Primate Research Center, Texas Biomedical Research Institute, San Antonio, TX 78227, USA

**Keywords:** β-carotene, lycopene, β-cryptoxanthin, α-carotene, retinol, tocopherol, lutein, zeaxanthin, after-school, community programming

## Abstract

Over 85% of young women in the United States do not meet fruit and vegetable intake recommendations, placing them at risk for low antioxidant nutrient intake. Social health programming (SHP) can improve dietary intake of fruits and vegetables, but it is not known how SHP impacts serum levels of specific antioxidant nutrients. This observational cohort study assessed the effect of participation in SHP through Girls Inc., Omaha, on serum carotenoid, retinol, and tocopherol levels for 12–29-year-old women. Serum nutrient levels were measured using high-performance liquid chromatography and nutrient intake from diet was measured using three 24 h dietary recalls (ASA24^®^). Pearson chi-squared tests, Mann–Whitney U tests, and linear regressions were used to compare differences in nutritional status between SHP participants and non-participants. After adjustment for age and race/ethnicity, SHP participation was associated with significantly higher serum concentrations of total lycopene, δ-tocopherol, β-carotene, β-cryptoxanthin, lutein + zeaxanthin, and α-carotene. There were no between-group differences in average daily intake of carotenoids, vitamin A, or vitamin E after adjustment for race/ethnicity and age. These findings suggest that SHP may be a successful intervention to improve antioxidant nutritional status.

## 1. Introduction

Antioxidant nutritional status is intimately tied to a myriad of health outcomes including growth and development throughout childhood and maternal and infant wellbeing during pregnancy [1,2,3,4]. Nutrients that act as antioxidants such as carotenoids, vitamin A, and vitamin E may be particularly important in reducing oxidative stress and promoting optimal health prior to pregnancy [1,5]. However, nearly 90% of adults in the United States do not meet recommended intake levels for fruits and vegetables [6], placing them at risk for lower dietary antioxidant capacity and lower plasma levels of carotenoids [7]. Additionally, these critical nutrients may be difficult to obtain for women who report food insecurity or reside in a food desert, both of which are characterized by limited access to fresh produce [8,9,10]. Socioeconomic disparities in carotenoid and retinol nutritional status have also been documented, emphasizing the importance of nutritional interventions for girls and young women as part of a solution to mitigate nutrition-associated maternal health disparities [9,11].

Social health programming (SHP) is a multifaceted community-driven intervention that empowers communities to address modifiable risk factors for poor health, including suboptimal nutrition. Although SHP is tailored to fit the needs of individual communities, hallmarks of SHP include engaging health and nutrition education, mentorship and internship opportunities, and exploration of personal interests such as gardening, cooking, and art [12]. Nutritionally focused SHP can provide tangible health benefits for participants such as increased fruit and vegetable intake, improved self-efficacy with cooking healthy meals, and decreased percent body fat [13,14,15,16,17,18]. One study found that fruit and vegetable consumption during the first year of an SHP intervention was a significant predictor of fruit and vegetable consumption 5 years later [19], highlighting that early SHP participation can provide long-term nutritional benefits and has the potential to impact dietary antioxidant capacity.

Although SHP participation has been shown to improve dietary quality [13,14,15,16,17,18], the impact of SHP participation on serum levels of antioxidant nutrients remains unknown. To fill this gap in the scientific literature, we conducted an observational cohort study to determine how SHP participation during adolescence impacts serum levels of carotenoids, vitamin A, and vitamin E.

## 2. Materials and Methods

### 2.1. Partnership with Girls Inc., Omaha

Girls Inc. is an international organization providing SHP to girls in over 350 cities across the United States and Canada. Each Girls Inc. location is dedicated to inspiring all girls to be strong, smart, and bold by providing them with the resources and social support they need to reach their full potential. Girls Inc., Omaha, is an exemplary local branch of the national Girls Inc. organization, providing SHP in Omaha, Nebraska. Programming is offered 5 days per week, with after-school programming provided during the school year and full-day programming during school holidays. Programming is provided by Girls Inc. staff as well as community partners. Activities range from school tutoring and athletics to mindfulness training and health education. Nutrition-focused programming includes gardening, aquaponics, and cooking classes in a fully equipped teaching kitchen. These activities encourage girls to increase their fruit and vegetable consumption by familiarizing girls with the produce they grow and providing opportunities to cook and eat fruit and vegetable dishes. Additionally, a fruit or vegetable is provided at every meal for girls who elect to eat the lunch and/or dinner provided by Girls Inc.

Nebraska Medicine and the University of Nebraska Medical Center have an established partnership with Girls Inc., Omaha. Nebraska Medicine maintains a clinic at Girls Inc. and hosts a variety of summer internships, career exploration days, and other events in partnership with Girls Inc., Omaha. Research personnel for this study provided additional monthly programming, including nutrition education games and produce-focused cooking activities such as making eggplant parmesan and blueberry zucchini bread.

### 2.2. Study Design and Participant Eligibility

This observational cohort study was approved by the University of Nebraska Medical Center Institutional Review Board (IRB# 0241-21-FB). A parent/guardian of all minor participants (≤18 years old) provided written informed consent prior to participation. The minor participants also provided written assent prior to participation. All adult participants provided written consent prior to participation.

Participants in the SHP group were defined as women who had participated in Girls Inc., Omaha, prior to study enrollment. Participants in the control group were defined as women who had never participated in any SHP. Blood samples for serum antioxidant levels, the primary study outcome, were collected during a single visit. Dietary intake of antioxidants, the secondary outcome, was assessed during all 3 study visits.

Eligible participants included 12–29-year-old females living in an area served by Girls Inc., Omaha (North or South Omaha). Women were excluded from this study if they participated in SHP through organizations other than Girls Inc., had a medical condition that would significantly alter absorption or metabolism of fat-soluble nutrients (such as cystic fibrosis with pancreatic insufficiency, inflammatory bowel disease, or a congenital metabolic disorder), or had a medical condition that would significantly alter normal cardiovascular homeostasis (such as a congenital heart defect, chronic kidney disease, or pheochromocytoma). After 1 year of recruitment, it was noted that participants in the control group were significantly more likely to self-identify as non-Hispanic White compared to participants in the SHP group. For the remainder of the enrollment period, individuals who did not attend Girls Inc. and self-identified as non-Hispanic White were also excluded from participation to ensure that study groups were demographically matched. 

### 2.3. Participant Recruitment

Participants were enrolled between March 2022 and May 2024. Research personnel discussed the study in person with potential participants at community health fairs and monthly events at Girls Inc. Additionally, fliers were posted on a variety of platforms including the Girls Inc. digital newsletter, Facebook and Instagram, and community centers such as grocery stores and churches. A total of 458 individuals expressed interest in research participation. Of these interested individuals, 209 declined to complete eligibility screening, 140 did not meet inclusion/exclusion criteria, 11 declined to participate after eligibility screening, 10 withdrew prior to completing any study visits, and 7 were lost to follow up without completing study activities to assess dietary intake or serum levels of antioxidant nutrients. Twenty-five SHP participants and fifty-six participants in the control group completed the study and were included in this analysis.

### 2.4. Study Questionnaires

All questionnaires, except for the Automated Self-Administered 24-Hour (ASA24^®^ version 2022 US) Dietary Assessment Tool, were collected and managed using REDCap (version 17.0.1) electronic data capture tools hosted at the University of Nebraska Medical Center. A household sociodemographic questionnaire and the validated 18-item US Household Food Security Survey Module were completed by adult study participants (≥19 years old) or a parent/guardian of participants less than 19 years old [20]. Collected information included self-reported barriers to obtaining healthy foods, access to supplemental nutrition programs (SNAP or WIC), estimated annual household income, and number of people in the home. An income-to-poverty ratio was calculated by dividing the annual household income by the 2023 federal poverty guideline for the reported household size [21].

Both adult and minor participants completed 3 validated ASA24^®^ questionnaires and a health questionnaire [22]. The ASA24^®^ uses digital photos as portion size estimation aids for most foods, which have been shown to improve dietary intake estimates in children [23]. Nutrient intake from 3 ASA24 dietary recalls was averaged to calculate the estimated daily intake per 1000 kcal. Participants who completed less than 3 ASA24 dietary recalls were excluded from dietary intake analysis. All participants completed at least 1 ASA24 in-person with study personnel present to answer any questions. Participants’ vitamin A and vitamin E intake was categorized as adequate or inadequate according to the Food and Nutrition Board at the Institute of Medicine [24,25]. Information collected in the health questionnaire included height and weight, nicotine use, and self-reported race/ethnicity. Additionally, SHP participants self-reported the number of years they participated in Girls Inc., Omaha, how many months per year they participated, and how many days per week they participated.

### 2.5. Serum Nutrient Analysis

Study personnel trained in phlebotomy collected approximately 3 mL of whole blood in serum separator tubes or serum tubes. Blood samples were protected from heat and light prior to centrifugation for 4 min at 3200 RPM. The resultant serum was aliquoted and frozen at −80 °C within 3 h of sample collection.

Serum aliquots were analyzed for retinol, tocopherol (α, γ, and δ) and carotenoid (α-carotene, β-carotene, lutein and zeaxanthin, lycopene, and β-cryptoxanthin) concentrations using reverse-phase high-performance liquid chromatography at the Nutritional Biomarker Lab (Harvard T.H. Chan School of Public Health), as previously described [26,27]. Serum samples (250 μL) were mixed with 250 mL ethanol containing 10 μg racemic tocopherol/mL (Tocol; Matreya Inc., Pleasant Gap, PA, USA) as an internal standard, extracted with 4 mL hexane, evaporated to dryness under nitrogen at 20 °C, and reconstituted in 100 mL ethanol:dioxane (1:1, by volume) and 150 mL acetonitrile. The extracted samples were saponified with potassium hydroxide for 15 min at 50 °C. Before saponification, 500 μL of 20% (weight:volume) ascorbic acid in aqueous solution and 1000 μL of 0.167 μg trans-β-apo-8′-carotenal/mL in ethanolic solution were added to all samples. Concentrations of the antioxidants were then quantitated by HPLC on a Restek Ultra C18 150 mm × 4.6 mm column with a 3 μm particle size (Restek Corp, Bellefonte, PA, USA). The column temperature was 20 °C and the room temperature was 22–23 °C. The column was encased in a water bath to prevent temperature fluctuations and was equipped with a trident guard cartridge system. A mixture of acetonitrile, tetrahydrofuran, methanol, and a 1% ammonium acetate solution (68:22:7:3) was used as the mobile phase with a flow rate of 1.1 mL/min. The system included a Hitachi L-7100 pump in isocratic mode, an L-4250 ultraviolet–visible light (445 nm) detector, and a programmable AS-4000 autosampler with a water-chilled tray interfaced with a D-6000 interface module (Hitachi, San Jose, CA, USA). The system manager software (D-7000, version 3.0; Hitachi) was used for peak integration and data acquisition. The minimum detection limits in plasma were 7.74 μg/L for α-carotene, 7.31 μg/L for β-carotene, 5.51 μg/L for β-cryptoxanthin, 8.49 μg/L for lycopene, and 6.31 μg/L for lutein + zeaxanthin.

### 2.6. Statistical Analysis

Statistical analyses were completed using IBM SPSS software version 27. Medians and interquartile ranges were calculated for continuous variables while frequencies and percentages were calculated for categorical variables. Mann–Whitney U tests were utilized to compare continuous nutrient serum and intake levels between groups (SHP vs. control). Pearson chi-squared tests were used to compare categorical variables between groups. Directed acyclic graphing was utilized to identify age and race/ethnicity as potential confounders [9,28,29]. Multiple linear regressions were used to predict nutrient serum concentrations or average daily intake based on the following independent variables: SHP participation (control group vs. SHP participant), race/ethnicity (non-Hispanic Black vs. non-Hispanic White vs. other race/ethnicity), and age (continuous). Participants were only excluded from analyses for which they had incomplete data.

## 3. Results

### 3.1. Participant Demographics

SHP participants attended Girls Inc., Omaha, for a median of 4 years (IQR 2.0–5.8) prior to participating in this study. Participants attended Girls Inc. a median of 4 days per week (IQR 1.5–5.0) during the academic school year and 5 days per week (IQR 3.0–5.0) during the summer months. At the time of study participation, 40% of SHP participants were currently attending Girls Inc., Omaha, and 60% were previous attendees. Among the previous attendees, the last participation in Girls Inc. occurred a median of 4 years prior to the study (IQR 2.0–8.0).

With the exception of age (*p* = 0.01) and race/ethnicity (*p* < 0.001), participant sociodemographic characteristics were similar between the SHP and control groups. There was no significant difference between groups in measures of food access, including household food security, self-reported barriers to obtaining healthy foods, and SNAP/WIC participation (Table 1). The most common self-reported barriers to obtaining healthy foods were cost (12.0% of SHP participants and 16.1% of control group) and storage (4.0% of SHP participants and 8.9% of control group).

### 3.2. Dietary Intake

Dietary intake information was available for 23 SHP participants and 47 participants in the control group. There were no between-group differences in the proportion of SHP participants who met dietary intake recommendations for vitamin A (13.0% vs. 23.4%; *p* = 0.31) or vitamin E (0% vs. 6.4%; *p* = 0.22) compared to participants in the control group. Similarly, there were no significant differences between groups in dietary intake of α-tocopherol, retinol, or the carotenoids assessed in this study (Table 2). However, SHP participants did have a significantly lower median intake of vitamin A compared to participants in the control group. After adjustment for age and race/ethnicity, dietary intake of vitamin A was similar between groups (β = −65.42, *p* = 0.08, and 95% CI −138.53 to 7.69).

### 3.3. Serum Carotenoid Concentrations

**Figure 1:**
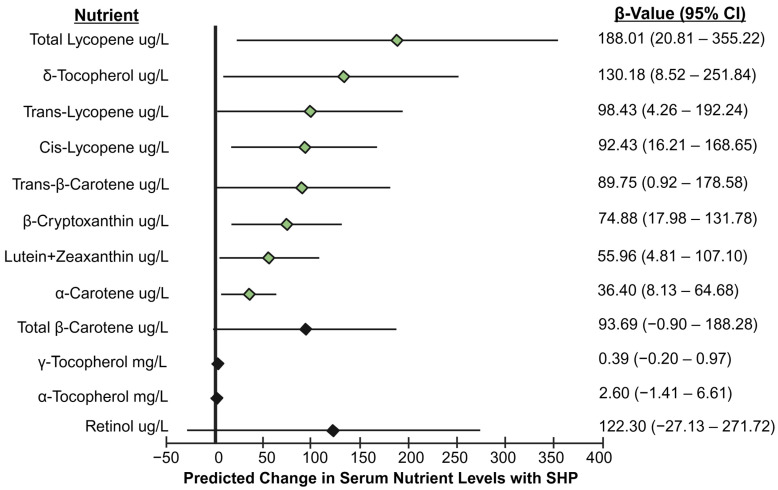
Predicted increases in serum nutrient concentrations associated with SHP participation after adjustment for race/ethnicity and age. Lines represent 95% confidence intervals and diamonds represent the β-values from multiple linear regression models. Significant models are noted with a green diamond and non-significant models are shown with a black diamond. Created in BioRender. Drakowski, R. (2025) https://BioRender.com/u20h028.

Serum nutrient concentrations were available for 16 SHP participants and 42 participants in the control group. SHP participants had significantly higher median serum concentrations of lutein + zeaxanthin; β-cryptoxanthin; and trans-, cis-, and total lycopene. There was no significant between-group difference in serum α-carotene, trans-β-carotene, or total β-carotene (Table 3). After adjustment for age and race/ethnicity, SHP participation was associated with significantly higher serum concentrations of trans-β-carotene; α-carotene; β-cryptoxanthin; lutein + zeaxanthin; and trans-, cis-, and total lycopene (Figure 1).

### 3.4. Serum Vitamin A and E Concentrations

SHP participants had significantly higher median serum concentrations of retinol, α-tocopherol, and δ-tocopherol compared to the control group. There was no significant difference between groups in serum γ-tocopherol (Table 3). After adjustment for age and race/ethnicity, SHP participation continued to be associated with a significantly higher δ-tocopherol serum concentration (Figure 1).

## 4. Discussion

Our study revealed that SHP participants had significantly higher serum levels of δ-tocopherol and multiple carotenoids compared to the control group after adjustment for relevant confounders. As plasma concentrations of certain carotenoids are validated biomarkers of fruit and vegetable intake [30], our findings align with previous studies reporting that participation in nutrition-focused SHP is associated with increased intake of fruits and vegetables [13,14,15]. For example, Jarpe-Ratner et al. found that 3rd–8th grade children significantly increased their fruit and vegetable consumption following a 10-week nutrition intervention and cooking class [13]. Similarly, participation in a youth gardening program was associated with increased fruit and vegetable intake for 71% of studies included in a systematic review by Savoie-Roskos et al. [15]. The emphasis of SHP on increasing fruit and vegetable intake may explain why SHP participants had significantly higher serum levels of carotenoids after adjustment for confounders but no difference in retinol or α-tocopherol serum levels. Major dietary sources of carotenoids include leafy green, yellow, orange, and red fruits and vegetables, although carotenoids can be found in other foods including vegetable oils and eggs [24]. In contrast, major dietary sources of retinol and tocopherol include fish, eggs, nuts, and oils in addition to fruits and vegetables [24,25].

In this study, there were no significant differences in reported dietary intake of carotenoids, vitamin A, or vitamin E between the SHP and control groups. These findings are interesting given the increased serum carotenoid and tocopherol levels observed among SHP participants in this study, as well as the multiple previous studies which have shown that SHP participation is associated with increased consumption of antioxidant-rich fruits and vegetables [13,14,15]. Dietary intake was measured using three 24 h dietary recalls with digital two-dimensional portion size estimation aids for most foods, which have been extensively validated in a United States population [22]. However, previous studies reveal that children and young adolescents are more prone to errors in dietary recall compared to older adolescents or adults [31,32]. As our SHP participants were significantly younger than participants in the control group, it is possible that the reported dietary intake was less accurate for SHP participants compared to the control group despite adjustment for age in multiple linear regression models. The direction of error (overestimation vs. underestimation) varies across studies [23]; for this study, either underestimation of nutrient-rich foods and/or overestimation of energy-dense foods among SHP participants could produce similar nutrient intake estimates between groups.

Alternatively, SHP participants could have higher serum concentrations of carotenoids and δ-tocopherol despite similar dietary intake if SHP participants were experiencing lower levels of oxidative stress compared to participants in the control group. Multiple factors can increase production of reactive oxygen species, free radicals, and other molecules that contribute to oxidative stress, including long-term stress exposure, smoking, and obesity [33]. Nutrients that act as antioxidants, including carotenoids, are metabolized during the process of neutralizing reactive oxygen species and free radicals [34]. Therefore, if participants in the control group had a higher level of oxidative stress compared to SHP participants, serum carotenoid and tocopherol levels could be depleted more rapidly among participants in the control group, resulting in lower serum nutrient concentrations. Although this study did not assess serum markers of oxidative stress, we did assess smoking status and BMI, two key factors associated with increased oxidative stress [35]. There were no differences between groups in the proportion of participants who were current smokers or had an obese BMI.

This study was limited by a small sample size recruited from a single local organization providing SHP (Girls Inc., Omaha). Recruitment for SHP participants was lower than anticipated, due in part to a reduction in the adolescent participation at Girls Inc., Omaha, following the COVID-19 pandemic. The difference in group sizes, with 25 SHP participants and 56 participants in the control group, could have impacted the statistical analysis of the results. Future studies should include current and former SHP participants from multiple organizations providing SHP to maximize sample size and ensure that study results are generalizable for adolescents and young women across the United States. Increasing sample size may also allow for separate analysis of current and former SHP participants, which could clarify the long-term impact of SHP on nutrition. Additionally, SHP participants and participants in the control group were not demographically matched by age or race/ethnicity, despite efforts to recruit a matched and balanced control group. We adjusted for age and race/ethnicity during statistical analysis to minimize the impact of this limitation on our study results. However, we acknowledge that due to the limitations of this study, the presented findings should be considered preliminary.

## 5. Conclusions

Although previous studies have found that SHP participation is associated with increased dietary intake of fruits and vegetables, our study is among the first to demonstrate the impact of SHP on serum concentrations of dietary antioxidants. We found that SHP participation for young women was associated with higher serum concentrations of carotenoids and δ-tocopherol. These results suggest that participation in nutrition-focused SHP may improve antioxidant nutritional status. Future studies should investigate the long-term impact of SHP participation on oxidative stress and antioxidant capacity.

## Figures and Tables

**Table 1:** Participant demographics by group.

	SHP Participant Median (IQR)	Control Group Median (IQR)	*p*-Value
Age (Years) ^1^	15.0 (13.0–17.0)	21.0 (15.0–25.8)	0.01 *
Income:Poverty Ratio ^1,2^	1.85 (0.95–2.87)	1.71 (0.91–3.42)	0.76
	**SHP Participant** **Percent (Count)**	**Control Group** **Percent (Count)**	***p*** **-Value**
Race/Ethnicity ^3^ Non-Hispanic Black Non-Hispanic White Other Race/Ethnicity Declined	**60.0 (15)** **16.0 (4)** **20.0 (5)** **4.0 (1)**	**10.7 (6)** **35.7 (20)** **50.0 (28)** **3.6 (2)**	<0.001 *
Nicotine Use ^3^ Never Used Current or Former Use	**84.0 (21)** **16.0 (4)**	**75.0 (42)** **25.0 (14)**	**0.30**
BMI ^3^ Healthy Weight Overweight Obese Declined	**24.0 (6)** **20.0 (5)** **32.0 (8)** **24.0 (6)**	**32.1 (18)** **17.9 (10)** **41.1 (23)** **8.9 (5)**	**0.83**
Household Food Security ^3^ High Marginal, Low, or Very Low Declined	**32.0 (8)** **64.0 (16)** **4.0 (1)**	**44.6 (25)** **56.4 (31)** **0.0 (0)**	**0.30**
Barriers to Obtaining Healthy Food ^3^ Any Barrier No Barrier Declined	**12.0 (3)** **80.0 (20)** **8.0 (2)**	**23.2 (13)** **76.8 (43)** **0.0 (0)**	**0.31**
SNAP/WIC Benefits ^3^ Yes No Declined	**24.0 (6)** **64.0 (16)** **12.0 (3)**	**25.0 (14)** **73.2 (41)** **1.8 (1)**	**0.87**
Education ^3,4^ High School or Less Some College College Graduate Declined	**32.0 (8)** **28.0 (7)** **32.0 (8)** **8.0 (2)**	**21.5 (12)** **37.5 (21)** **41.1 (23)** **0.0 (0)**	**0.46**
Marital Status ^3,4^ Single Married/Partnered Divorced/Separated/Widowed Declined	**32.0 (8)** **32.0 (8)** **28.0 (7)** **8.0 (2)**	**44.6 (25)** **42.8 (24)** **12.5 (7)** **0.0 (0)**	**0.17**
Employment ^3,4^ Full- or Part-Time Retired/Unemployed Declined	**64.0 (16)** **28.0 (7)** **8.0 (2)**	**82.1 (46)** **17.8 (10)** **0.0 (0)**	**0.22**

^1^ Mann–Whitney U tests were used to compare median values between groups. ^2^ Data were available for 21 SHP participants and 55 participants in the control group. ^3^ Pearson chi-squared tests were used to compare proportions between groups. Participants who declined to answer were excluded from analysis. ^4^ Data reported for adult participants or the parent/guardian of participants ≤ 19 years old. * *p*-value < 0.05.

**Table 2:** Average daily dietary intake per 1000 kcal.

	SHP Participant Median (IQR)	Control Group Median (IQR)	*p*-Value ^1^
Total Vitamin A (µg RAE)	212.97 (154.91–259.51)	291.78 (203.33–378.77)	0.02 *
Retinol (µg)	178.80 (89.99–239.40)	201.91 (144.32–282.78)	0.17
β-Carotene (µg)	308.49 (161.80–741.99)	699.08 (168.32–1060.57)	0.17
α-Carotene (µg)	22.55 (5.15–129.75)	27.22 (8.28–264.72)	0.16
β-Cryptoxanthin (µg)	17.76 (7.36–45.08)	12.58 (7.27–38.87)	0.54
Lycopene (µg)	1829.01 (684.87–4005.11)	1145.16 (415.72–3630.38)	0.29
Lutein + Zeaxanthin (µg)	273.05 (203.03–648.92)	431.70 (289.65–778.61)	0.23
α-Tocopherol (mg)	4.27 (3.32–4.77)	4.09 (3.48–5.05)	0.70

^1^ Mann–Whitney U tests were used to compare median values between groups. * *p*-value < 0.05.

**Table 3:** Serum concentrations of retinol, carotenoids, and tocopherol.

	SHP Participant Median (IQR)	Control Group Median (IQR)	*p*-Value ^1^
Retinol (µg/L)	506.18 (393.69–560.17)	319.15 (162.25–529.01)	0.03 *
Trans-β-carotene (µg/L)	122.48 (94.91–212.09)	77.75 (47.50–158.40)	0.10
Total β-carotene (µg/L)	124.06 (95.96–219.28)	79.50 (59.17–170.77)	0.13
α-Carotene (µg/L)	39.68 (22.01–74.38)	24.36 (2.31–43.01)	0.08
β-Cryptoxanthin (µg/L)	148.51 (100.25–222.65)	92.46 (43.40–166.93)	0.02 *
Trans-lycopene (µg/L)	245.12 (100.27–422.90)	91.20 (61.95–179.30)	0.003 *
Cis-lycopene (µg/L)	201.99 (97.85–336.56)	90.18 (37.88–165.61)	0.003 *
Total lycopene (µg/L)	446.69 (206.97–754.71)	175.38 (106.91–348.48)	0.003 *
Lutein + Zeaxanthin (µg/L)	194.46 (140.48–237.05)	134.58 (87.33–216.36)	0.03 *
α-Tocopherol (mg/L)	11.98 (9.23–15.72)	8.97 (4.53–13.41)	0.03 *
γ-Tocopherol (mg/L)	1.84 (1.41–2.27)	1.40 (0.92–2.18)	0.20
δ-Tocopherol (µg/L)	404.99 (233.39–507.10)	41.31 (41.31–409.31)	0.002 *

^1^ Mann–Whitney U tests were used to compare median values between groups. * *p*-value < 0.05.

## Data Availability

The raw data supporting the conclusions of this article will be made available by the authors on request.

## Abbreviations

The following abbreviations are used in this manuscript:

SHP	Social Health Programming
ASA24	Self-Administered 24-Hour (ASA24^®^) Dietary Assessment Tool
IQR	Interquartile Range
µg RAE	µg Retinol Activity Equivalent
